# IRBIS: a systematic search for conserved complementarity

**DOI:** 10.1261/rna.045088.114

**Published:** 2014-10

**Authors:** Dmitri D. Pervouchine

**Affiliations:** 1 Centre for Genomic Regulation and UPF, Barcelona 08003, Spain; 2 Faculty of Bioengineering and Bioinformatics, Moscow State University, 119992 Moscow, Russia

**Keywords:** RNA–RNA interaction, evolutionary conservation, long-range RNA structure, exon skipping, alternative splicing, Ca-α1D, Dystonin, snoRNA, lncRNA

## Abstract

IRBIS is a computational pipeline for detecting conserved complementary regions in unaligned orthologous sequences. Unlike other methods, it follows the “first-fold-then-align” principle in which all possible combinations of complementary *k*-mers are searched for simultaneous conservation. The novel trimming procedure reduces the size of the search space and improves the performance to the point where large-scale analyses of intra- and intermolecular RNA–RNA interactions become possible. In this article, I provide a rigorous description of the method, benchmarking on simulated and real data, and a set of stringent predictions of intramolecular RNA structure in placental mammals, drosophilids, and nematodes. I discuss two particular cases of long-range RNA structures that are likely to have a causal effect on single- and multiple-exon skipping, one in the mammalian gene *Dystonin* and the other in the insect gene *Ca-α_1_D*. In *Dystonin*, one of the two complementary boxes contains a binding site of *Rbfox* protein similar to one recently described in *Enah* gene. I also report that snoRNAs and long noncoding RNAs (lncRNAs) have a high capacity of base-pairing to introns of protein-coding genes, suggesting possible involvement of these transcripts in splicing regulation. I also find that conserved sequences that occur equally likely on both strands of DNA (e.g., transcription factor binding sites) contribute strongly to the false-discovery rate and, therefore, would confound every such analysis. IRBIS is an open-source software that is available at http://genome.crg.es/~dmitri/irbis/.

## INTRODUCTION

RNA–RNA interactions (RRIs) received increasing attention in recent years, especially in the light of growing evidence for abundant expression of noncoding RNAs (Ponting et al. 2009; Derrien et al. 2012). One current hypothesis is that RRI could specifically guide some of the regulatory programs in the RNA processing pathway, similar to what small RNAs do in the post-transcriptional gene silencing and translational attenuation. RRI plays a fundamental role in the functioning of the spliceosome, where small nuclear RNAs (snRNAs) interact with each other and with the pre-mRNA by forming hetero-duplexes (Will and Luhrmann 2011). Not only snRNAs do this; for instance, the C/D box snoRNA HBII-52 contains a sequence that is complementary to HT_2*C*_R mRNA and affects alternative splicing in this disease-associated gene (Kishore and Stamm 2006).

The problem of RRI prediction is technically very similar to RNA secondary structure (RSS) prediction, with the major difference being that base pairs both within and between RNA molecules are allowed. Although intra- and intermolecular interactions are driven by the same molecular forces, this distinction is crucial for algorithms because RSS is historically assumed to be nested (i.e., unknotted; see discussed below), while simultaneous prediction of intra- and intermolecular base-pairings is equivalent to RNA folding with pseudoknots (Pervouchine 2004; Alkan et al. 2006; Huang et al. 2009). Here I discuss the two problems jointly without assuming that RSS is nested and, in particular, ascribe long-range intramolecular base-pairings also to RRI.

Both RRI and RSS predictions comprise a broad range of methods that admit single-sequence (de novo) and multiple-sequence (comparative) formulations. Most of the de novo methods are based on thermodynamic energy model, which assumes additive contributions to the free energy function from elementary structural units (Mathews et al. 1999). However, the optimization method that is used to find the minimum free energy (dynamic programming) is computationally efficient only for nested RSS: For arbitrary pseudoknots, it is NP complete (Lyngsø and Pedersen 2000), and even for the most generic type of pseudoknots, the required time is *O*(*n*^6^) (Rivas and Eddy 1999). Besides this technical limitation, there is a fundamental problem that the additive model is insufficient to describe entropy contribution of loops in molecules with pseudoknots and that important steric and topological limitations also need to be taken into account (Pervouchine 2004). Consequently, RRI prediction methods avoid intramolecular interactions to be computationally efficient (Mückstein et al. 2006; Wenzel et al. 2012). The best current trade-off approach uses precomputed accessibility profiles in addition to free energy scoring of exposed binding sites (Mückstein et al. 2006; Tafer et al. 2011). Some methods gain additional speed by simplifications to the free energy model, which makes them practicable on a genome-wide scale as, for instance, microRNA target finders, although elimination of the internal RNA structure results in a dramatic increase of false-positive predictions (Rehmsmeier et al. 2004; John et al. 2006; Ragan et al. 2009).

In contrast, comparative methods take advantage of the evolutionary information to reduce the false-positive rate (Gardner and Giegerich 2004). Simultaneous alignment and folding, known as the Sankoff algorithm, is computationally overexpensive (Sankoff 1985). Instead, most existing methods take a so-called “first-align-then-fold” route in which a multiple sequence alignment (MSA) is analyzed, for instance, as a profile by a single-sequence algorithm (Seemann et al. 2010, 2011; Li et al. 2011) or by a probabilistic model (Knudsen and Hein 2003; Pedersen et al. 2006; Rivas et al. 2012). This approach strongly depends on the accuracy of MSA, and although some improvement can be achieved by considering suboptimal sequence alignments (Will et al. 2013), the major limitation remains that MSA does not always exist. The opposite, “first-fold-then-align” route has not been systematically investigated because optimal single-sequence predictions are not accurate enough to build a consistent alignment (Shapiro and Zhang 1990; Hochsmann et al. 2003; Gardner and Giegerich 2004).

Recent studies on RSSs in eukaryotic genes revealed widespread occurrence of long-range RRI with diverse functions such as riboswitches (Li and Breaker 2013) and mediators of exon skipping (Lovci et al. 2013), mutually exclusive exon choice (Kreahling and Graveley 2005; Yang et al. 2011), and other types of alternative splicing events (Raker et al. 2009; Pervouchine et al. 2012). Many of these structures are located in regions lacking reliable sequence alignments and contain long, ultraconserved stretches of complementary nucleotides, in which the interacting bases can be separated by distances as large as 10 kb. The analysis of such large fragments by the thermodynamic model is challenging in terms of both accuracy and speed (Will et al. 2007; Moretti et al. 2008; Harmanci et al. 2011; Wei et al. 2011). The accuracy is affected because long-range base-pairings become shunted by local nested structures as soon as dynamic programming is used for free energy minimization. The computational time of RNA folding is minimally cubic, unless the search is again constrained to local RSS. At this point, the question logically arises whether the thermodynamic model is, indeed, needed for long, continuous helices occurring in long-range eukaryotic RSS.

**FIGURE 1.**
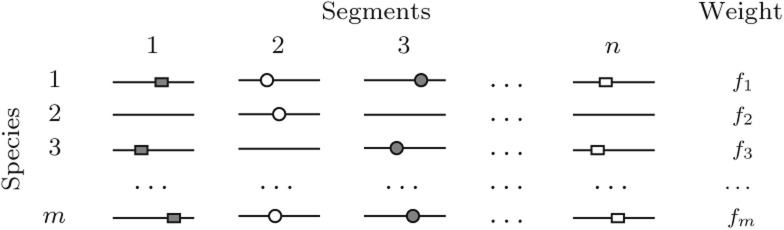
Orthologous segments *s*_*ij*_ are indexed by segment identifiers *j* = 1 … *n* in each of the species *i* = 1 … *m*. Gray boxes are complementary to white boxes and, respectively, gray circles to white circles. The positions of boxes and circles within segments do not play any role. All boxes and circles occur in orthologous segments in three species. Note, however, that boxes occur simultaneously in three species, while circles occur simultaneously in only two species.

This line of reasoning was elaborated in our recent reports on conserved long-range RSS in introns of mammalian and insect genes (Raker et al. 2009; Pervouchine et al. 2012). The strategy, which shares some technical ideas with GUUGle (Gerlach and Giegerich 2006), was to convert sequences into hash tables that store the location of each *k*-mer and to apply set-theoretic intersection (1) with the reverse complement and (2) across orthologs to detect instances of simultaneous complementarity and conservation. One important advantage of this method is that poorly conserved regions do not need to be aligned and, on the contrary, the lack of conservation becomes useful when assigning statistical significance to conserved “islands” immersed in a nonconserved intronic background (Raker et al. 2009). By construction, there are neither constraints on the distance between base pairs nor limitations on pseudoknots. This technique, in fact, applies to a broader range of settings than RSSs near splice sites. Figure 1 illustrates a general formulation in which the input is organized as a collection of unaligned orthologous sequence segments. In particular, such collection could consist of intronic windows adjacent to orthologous donor and acceptor splice sites or of orthologous miRNA precursors and 3′-UTRs in a miRNA target search, etc. The aim is to identify all pairs of complementary *k*-mers that occur in sufficiently many orthologous segments—no matter where, since the positions in unaligned sequences are not matched.

This setup is implemented here as a computational pipeline called IRBIS (intermolecular RNA interaction search, where “B” acknowledges a BLAT-like algorithm) (Kent 2002), which is designed to search for conserved, long-range RRI. The pipeline is fully automated and contains all necessary preprocessing steps, including genomic sequence download, identification of orthologous segments, selection of unique orthologs, etc. (Supplemental Material). Compared to the previous analyses (Raker et al. 2009; Pervouchine et al. 2012), which were restricted to short segments and to a relatively small number of their combinations, IRBIS has three major improvements: (1) the novel trimming procedure, which detects and removes a priori nonconserved *k*-mers; (2) gapped-seed hash tables for modeling short internal loops; and (3) sequence weighting. Of these, trimming is the essential speed-up step, allowing large-scale analyses. By default, IRBIS assumes that segments are defined by exon boundaries, but custom segmentations can also be used.

Since there are no methods that perform alignment-free RSS prediction without limitation on the distance between base pairs, IRBIS was compared to RNAplex (Tafer et al. 2011), currently the fastest RRI prediction tool, using (1) MSA generated by MUSCLE (Edgar 2004b) and (2) naïve MSA induced by IRBIS (see Materials and Methods). The relaxation of alignment constraints results in a higher discovery rate of IRBIS compared with RNAplex. At the same time, the predictions of the two programs are concordant when the latter is applied to a MSA generated by IRBIS. However, unlike other programs, IRBIS is able to find conserved complementary regions even in circumstances when MSA is not possible. By using IRBIS, I reexamined introns of mammalian, insect, and nematode protein-coding genes at much more depth than in previous reports and updated the lists of RSSs that are possibly implicated in splicing (Raker et al. 2009; Pervouchine et al. 2012). In particular, I discuss two specific cases of long-range RRI that are likely to cause single- and multiple-exon skipping (*Dystonin* and *Ca-α_1_D* genes). A systematic search for the potential snoRNA and lncRNA targets revealed that these two transcript classes have an increased capacity of base-pairing to introns of protein-coding genes. However, I also find a number of important confounding factors (such as transcription factor binding sites (TFBSs) that occur equally likely on both strands of DNA) that are maintained complementary by the evolution for purposes other than RRI. Altogether, these findings represent to date the most exhaustive large-scale analysis of conserved complementary motifs and reveal the applicability limits of comparative RRI prediction methods.

## RESULTS

This section is organized as follows. It begins with definitions and notation (the method itself is described in Methods section in the Appendix and the Supplemental Information). The method is benchmarked in several aspects. First, I demonstrate that IRBIS can find conserved complementary regions when orthologous segments are not alignable. Second, I compare IRBIS to RNAplex (Tafer et al. 2011), currently the fastest tool to predict intramolecular RRI, on MSA generated by MUSCLE (Edgar 2004b) using simulated data. I then apply IRBIS to intramolecular RSS prediction and follow a few specific cases, including RSSs in the mammalian *Dystonin* and in the insect *Ca-α_1_D* genes (Intramolecular RSS section). Next, I proceed to intermolecular RRI and perform a genome-wide search for snoRNA and long noncoding RNA (lncRNA) complementary targets (Intermolecular RRI section). I use the specific case of RP11-439A17.4 lncRNA to demonstrate an important artefact, in which a TFBS that occurs on opposite DNA strands is recognized as a conserved complementary trans-RRI. Next, I continue with the benchmark by comparing IRBIS and RNAplex on the set of naïve MSA generated by IRBIS to assess the similarity of the two programs in terms of base-pairings that they predict. Finally, I provide a short summary of the program performance (Resource Requirements) (see Table 2, below).

### Notation

The input to IRBIS consists of a collection of unaligned orthologous segments *s*_*ij*_ that are indexed by species *i* = 1 … *m* and segment *j* = 1 … *n* so that orthologous segments in different species *i* receive the same segment identifier *j* (Fig. 1). Species are given weight factors *f*_*i*_, *i* = 1 … *m*, which sum up to one. We are interested in finding short complementary words of length *k* that are conserved, that is, ones that occur in many *s*_*ij*_ for the same *i* (by default *k* = 8). One way to define “many” is to require that the sum of weights of the corresponding *s*_*ij*_ is greater than some threshold *t* (for details, see Materials and Methods). I require that complementary pairings contain at least *h* GC base pairs and allow up to *G* wobble GT base pairs. Complementary *k*-mers can overlap forming larger structures. I therefore cluster overlapping *k*-mers and require at least *L* complementary nucleotides in a cluster (for the complete list of parameters, see Table 1).

**TABLE 1.**
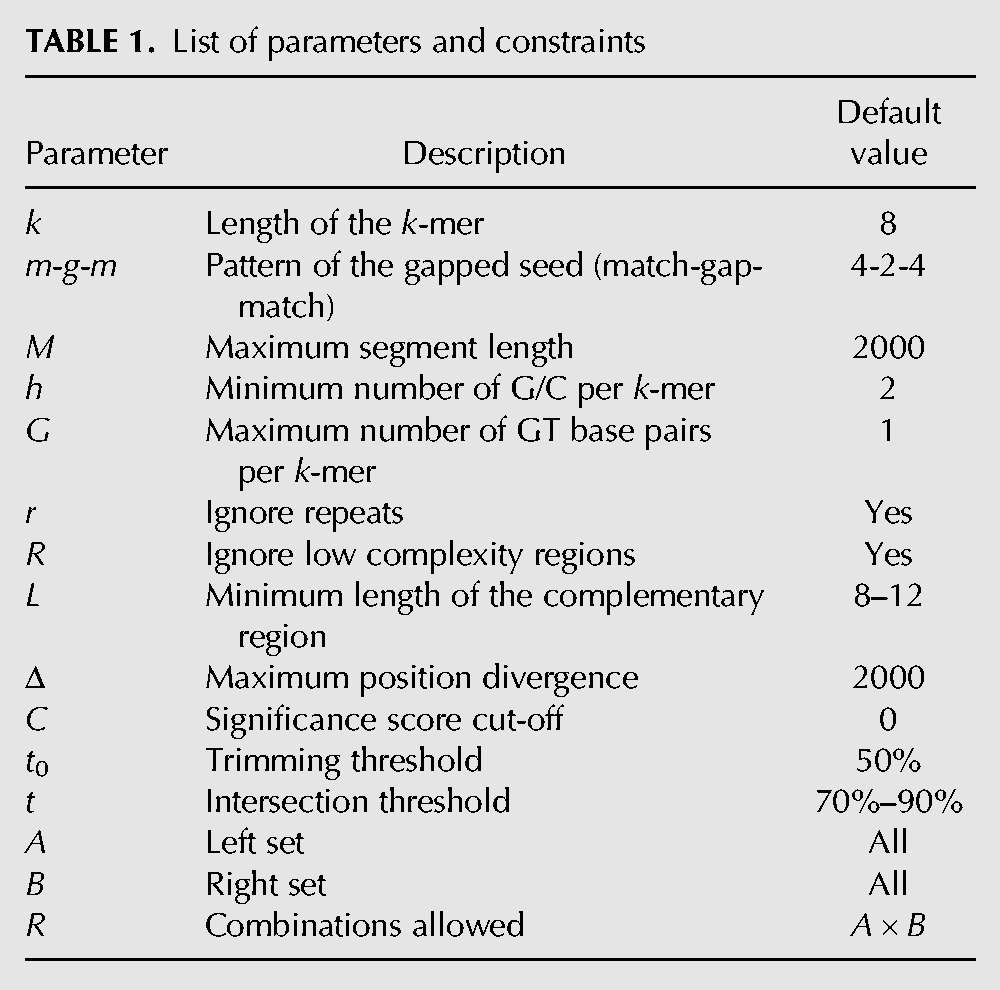
List of parameters and constraints

In different applications, it makes sense to search for complements between different sets of segments. I formalize this by searching for complementary matches between *s*_*aj*_, *a* ∈ *A* and *s*_*bj*_, *b* ∈ *B*, where *A* and *B* are (not necessarily disjoint) subsets of *i* = 1 … *m*. For instance, when searching for snoRNA targets, *A* is the set of snoRNA segments and *B* is the set of target segments. The number of all-to-all combinations of segments from *A* and *B* can sometimes be very large. I therefore confine my search to a subset of combinations defined by a relation ℛ between elements of *A* and *B*. For example, when searching for conserved RSS, both *A* and *B* are the set of intronic segments, but only segments from the same gene are allowed in the relation ℛ. More information on the notation can be found in the Materials and Methods section.

### Benchmarking

In order to assess sensitivity, I generated *n* = 200 random 1000-nt-long sequences (with equal nucleotide probabilities) independently in each of *m* = 16 simulated species, 100 random seed sequences of length *k* = 8, and inserted the seeds into the segments with odd numbers so that *s*_*ij*_ with the same *j* = 1, 3, 5 … received the same seed at independent uniformly distributed positions. Seeds' reverse complements were inserted also at uniformly random positions in segments with *j* = 2, 4, 6 … so that two consecutive segments, *s*_*ij*_ and *s*_*ij*__+1_, where *j* is odd, contained complementary seeds. Additionally, gaps of random size *l* ≤ 2 were introduced in the middle of each seed and in the middle of its reverse complement.

All 100 complementary seed pairs were detected in 100 independent trials at *t* = 95%. The sensitivity was also 100% at lower thresholds, with the exception of one case out of 100 when a random higher-scoring structure happened to occur in the same pair of sequences, *s*_*ij*_ and *s*_*ij*__+ 1_. At the same time, none of 200 groups of segments could be aligned because their sequences were completely unrelated. That is, the sensitivity of IRBIS is very high in the conditions where no other method can detect simultaneous complementarity; namely, if there is a pair of conserved complementary regions satisfying the requirements of Table 1, it will necessarily be detected.

In order to simulate random sequences that can be aligned, I generated *n* = 100 150-nt-long random sequences (nucleotide probabilities, 0.25) and applied evolutionary simulation on a binary tree with a nucleotide mutation rate of 3% to each sequence. As a result, I obtained *n* = 100 groups of orthologous segments with *m* = 16 segments in each group. Segments were aligned by MUSCLE software with the default settings (Edgar 2004b). Next, I analyzed all 4950 pairwise combinations with RNAplex (Tafer and Hofacker 2008). The predictions were filtered by requiring eight consecutive complementary nucleotides, at most one GT base pair per each 8 nucleotides (nt), and sequence conservation in 12 of 16 species. The same segments were analyzed by IRBIS with (no gaps, *k* = 8, *G* = 1, *t* = 0.75, *A* = *B* = {1,…, 100}, ℛ = *A* × *B*). RNAplex identified RRI in 30 pairs of segments, all of which were also identified by IRBIS, while IRBIS additionally found RRI in 71 other segments that were not identified by RNAplex. In all these 71 cases, the complementary *k*-mers were aligned differently by the two programs, indicating that the higher sensitivity of IRBIS is due to the relaxation of alignment constraints.

### Intramolecular RSS

According to the described formalism, IRBIS predicts intramolecular RRI when *A* and *B* are the same set of segments and the relation ℛ is such that (α, β) ∈ ℛ if α and β belong to the same gene. In the following analysis, I considered only noncoding segments of mammalian, insect, and nematode genes (350,000, 60,000, and 120,000 such segments, respectively). By using stringent thresholds, I obtained 832 pairs of complementary regions in 16 placental mammals (*L* = 12, *t* = 0.8), 632 pairs in 12 drosophilids (*L* = 12, *t* = 0.75), and 241 in six nematodes (*L* = 12, *t* = 0.6). Even at the most pessimistic FDR estimates of 30% (see below), hundreds of them are expected to be true positives. I ranked the predictions according to their significance score. The catalog and the automated alignments of these structures are listed in Supplemental Material. Below I estimate the false-discovery rate (FDR) and discuss a few specific examples, in which the mechanistic link between conserved RRI and molecular function appears to be evident.

### False-discovery rate

In order to estimate the FDR, I followed the rewiring control procedure described previously (Pervouchine et al. 2012). This procedure creates hybrid transcripts that consist of segments that belong to different genes but preserves dinucleotide composition and sequence conservation. It represents a good null model assuming that there are no RRI in *trans*. For mammalian genomes, the set *A* of 350,000 noncoding segments of human protein-coding genes was assigned into 500 blocks on the basis of segment's conservation score and GC content. The reference (true) sets of predictions were obtained by running IRBIS at variable *t* and *L* with *B* = *A* and ℛ such that (*j*, *j*′) ∈ ℛ if and only if *j* and *j*′ belong to the same gene (i.e., considering only intramolecular RNA structure). The relation ℛ contained about 3 million pairs of segments. The rewired control sets were obtained by running IRBIS at the identical conditions with the exception that ℛ was shuffled randomly, preserving the blocking by conservation and GC content (see Supplemental Methods). FDR was then defined to be the number of segment pairs in the control set as a fraction of the number of segment pairs in the reference set. The rewiring procedure was repeated 20 times to estimate the standard error of the mean.

**FIGURE 2.**
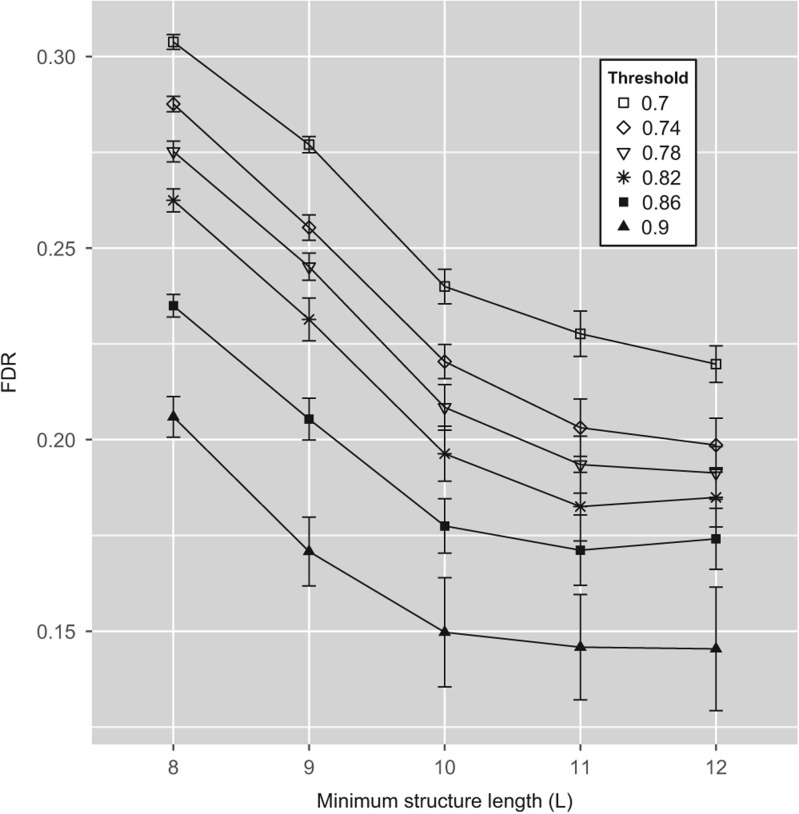
False-discovery rate (FDR) as a function of length threshold (L) and intersection threshold (t) for intramolecular RNA structure in noncoding segments of mammalian protein-coding genes. Error bars, 95% confidence intervals. Other parameters are as in Table 1.

Overall, the constraint on the length of the complementary region reduced FDR more significantly than did the constraint on the intersection threshold (Fig. 2). When no threshold on *L* was imposed (*L* = *k* = 8), FDR was >30%, in consistence with the figures reported previously (Pervouchine et al. 2012). The constraint of having complementary region of at least *L* = 12 nucleotides in combination with a high intersection threshold reduced FDR to ≈15%.

### Specific examples

**FIGURE 3.**
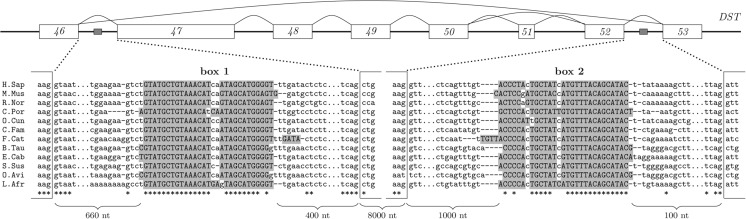
(*Top*) Exonic structure of a 9567-nt fragment of the human *DST* gene (Dystonin, Bullous Pemphigoid Antigen 1, BPAG1) on chr6:56,465,020–56,474,586. Exons 47–52 are spliced as a cluster. Two complementary sequences, box 1 and box 2, are located in introns between exons 46–47 and 52–53, as indicated by gray boxes. (*Bottom*) Multiple sequence alignment of introns containing boxes 1 and 2. The average sequence conservation rate is <0.5%.

The mammalian gene *Dystonin* (DST, BPAG1, bullous pemphigoid antigen) is a member of the plakin protein family that is involved in subepidermal skin blistering disease (Stanley 1993). A number of alternatively spliced isoforms have been reported for this gene (Okumura et al. 2002), including isoforms that encode plectin type of repeats (PTRs) and spectrin type repeats (STRs). In particular, exons 47–52 that encode a group of PTRs (Punta et al. 2012) are either spliced out or included as a cluster, with the exception of exon 51, which can be skipped independently (Fig. 3, BPAG1eA, top panel).

The human gene *Dystrophin* has a similar exonic architecture, where the most common type of multiple exon-skipping also affects the region encoding STRs (Roberts et al. 1993). Recent modeling studies reported that a protein with hybrid repeat types produced by multiple exon-skipping may have substantially different folding properties compared with the unskipped protein (Menhart 2006). Also, mammalian EST data suggest that splicing of the exon 47–52 cluster occurs differently in different tissues and at different developmental stages (Karolchik et al. 2003). Although it is not known what triggers multiple-exon skipping in this gene, the molecular mechanism is more likely to effectuate splicing of one long intron than that of seven consecutive shorter introns. But how do the flanking sequences, exons 46 and 53, recognize each other over a distance of ≈10,000 nt?

My analysis demonstrates that the intron downstream from exon 46 and one upstream of exon 53 contain a pair of complementary regions, box 1 and box 2, that are conserved across most of the placental mammals (Fig. 3). This pair of boxes escaped from our previous study (Pervouchine et al. 2012) because of its distant location: the distance distance between box 1 and the nearest exon boundary is >400 nt. Considering that the complementary sequences are ≈100% conserved, while the rest of the intronic sequence is not, it is quite plausible that this multiple exon-skipping event in *Dystonin* is mediated by the RNA structure, which encloses exons 47–52 in a loop when boxes 1 and 2 are paired. Notably, the conservation ends abruptly at the borders of the complementary regions (*P* = 10^−^^10^), indicating positive selection on the sequences of the boxes and on the complementarity between them despite the distance of ≈10 kb.

**FIGURE 4.**
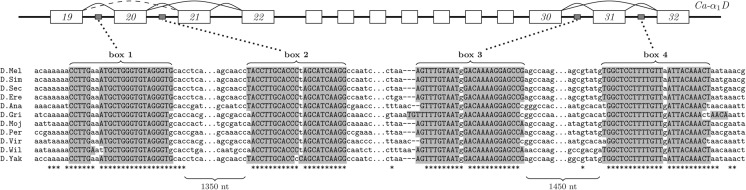
(*Top*) Exonic structure of Ca-α_1_D, a L-type voltage-gated calcium channel gene in the fruit fly (fragment chr2L:16,179,790–16,187,319). Exons 20 and 31 can be included or skipped. Although exon 19 is not annotated as a cassette exon, it can also be skipped in a tissue-specific way as evidenced by ESTs (dashed arc). (*Bottom*) Two pairs of very conserved complementary sequences, box 1/box 2 and box 3/box 4, found in introns surrounding these cassette exons (*bottom*) are likely to be involved in regulation of these splicing events.

Another example is the insect gene *Ca-α_1_D* that gives rise to L-type (high voltage activated) calcium channels (Fig. 4). Several transcript isoforms are annotated for this gene, ones in which exons 15 and 16 are mutually exclusive, exon 21 is skipped, or exon 31 is skipped. Additionally, EST data suggest that exon 20 can also be skipped (Karolchik et al. 2003). I find that the introns surrounding exons 20 and 31 contain two pairs of conserved complementary regions, box 1/2 and box 3/4, with high potential of base-pairing. The surrounding intronic sequences diverged almost completely, and the conservation of boxes wears off together with complementarity, suggesting that selection maintained these regions over 40 mya of evolution for base-pairing; it is very likely that the loops created by box 1/2 and box 3/4 serve as hallmarks for exon skipping. Additionally, a conserved hairpin structure is found at and downstream from the donor site of exon 5, although there is no evidence of alternative splicing or intron retention at this splice site (Supplemental File 1, p. 256).

Notably, the examples in Figures 3 and 4 are alternative splicing events; that is, they occur with different outcomes in different conditions. Therefore, there must be a mechanism that changes availability of complementary boxes, allowing the RNA structure to form and unform reversibly. I searched the doRiNA database of post-transcriptional regulatory elements (Anders et al. 2012) and found that there is iCLIP evidence of TIA-1 binding 16 nt upstream of box 2 of *Dystonin*. I therefore hypothesize that TIA-1 could be involved in the regulation of multiple exon-skipping in this gene. I also found that box 1 sequence in *Dystonin* gene contains the GCATG motif that has been shown to be a binding site of *Rbfox1*, which is also involved in exon skipping by forming a long-range RRI (Lovci et al. 2013).

### Sensitivity and false-negative rate

I next asked what is the sensitivity of IRBIS with respect to already known intronic RNA structures. Since there are only a few true positives, here I discuss them one by one. The box pairs in the *CG33298* and *Gug* genes were detected (Supplemental File 1, pp. 44, 476); the structure in the *Nmnat* gene could be detected only after relaxing the minimum length constraint to *L* = 10 (Raker et al. 2009). One of the structures predicted for the *SF1* gene was found (Supplemental File 2, p. 662), while other structures were detected at lower thresholds (Pervouchine et al. 2012). Interestingly, I find three previously unknown helices in the *Dscam* gene—two related to mutually exclusive exons 18 and 19 and one to exon 4 cluster (Supplemental File 1, pp. 35–37)—but none of the RNA structures that are responsible for mutually exclusive splicing in exon 4 cluster and exon 6 clusters. This is an expected result since regulatory RNA structures in *Dscam* are not universally conserved among Drosophilids (Yang et al. 2011). Following other examples by Yang et al. (2011), I found that the complementary regions IE1, IE2, and IEa, which mediate mutually exclusive splicing of exon 5 cluster in *14-3-3*ζ, were not detected despite high sequence conservation; the complementary regions in exon 7 and exon 11, but not in exon 9 cluster, of the *Mhc* gene were detected.

I looked deeper into *14-3-3*ζ exon 5 cluster predictions and found that pairwise sequence alignments mistakenly mapped IE1 to the intron upstream of, not downstream from, exon 5a. I successfully found conserved complementarity between IE1, IE2, and IEa after correcting pairwise sequence alignment. However, I also found a conserved 14-mer TTCACCAGCGAGGG in exon 5c that is complementary to a conserved 14-mer CCTTTGCTGGTGAA upstream of IEa, suggesting that the complete list of complementary boxes in this gene is to be continued.

### Intermolecular RRI

#### SnoRNA targets

Although the main function of snoRNA is to guide chemical modifications of other RNAs, some of them or their fragments can regulate splicing or translation (Kishore et al. 2010; Scott et al. 2012; Stepanov et al. 2012). While some (in particular, H/ACA box) snoRNAs interact with their rRNA targets by small bipartite recognition sites, the known cases of splicing regulation contain long, uninterrupted helices. A famous example is the HBII-52 (SNORD115) snoRNA that forms a perfect 18-nt helix with the human serotonin receptor mRNA and serves as a patch in splicing of this gene associated with Prader-Willi phenotype (Stepanov et al. 2012).

To investigate whether other RRIs between snoRNA and introns of mammalian protein-coding genes exist, I reconfigured the pipeline so that *A* and *B* were two disjoint sets: *A* was the set of 1500 segments of human snoRNA, and *B* was the set of 200,000 intronic noncoding segments of protein-coding genes (for definitions, see Supplemental Material). I used ℛ = *A* × *B* to account for all-to-all combinations of snoRNA from the set *A* with intronic sequences of protein-coding genes from the set *B*. Since the rewiring procedure was not applicable for this configuration, I used the control set *B*′ composed of sequences on the opposite strand from intronic segments of protein-coding genes. The advantage of this null model is that the sequences in *B*′ have exactly the same nucleotide conservation rate and GC content as sequences in *B*. However, if a snoRNA resides in an intron, this control procedure will always find it complementary to its own reverse complement. Therefore, introns containing annotated snoRNA have to be excluded from the set *B* (and *B*′).

**FIGURE 5.**
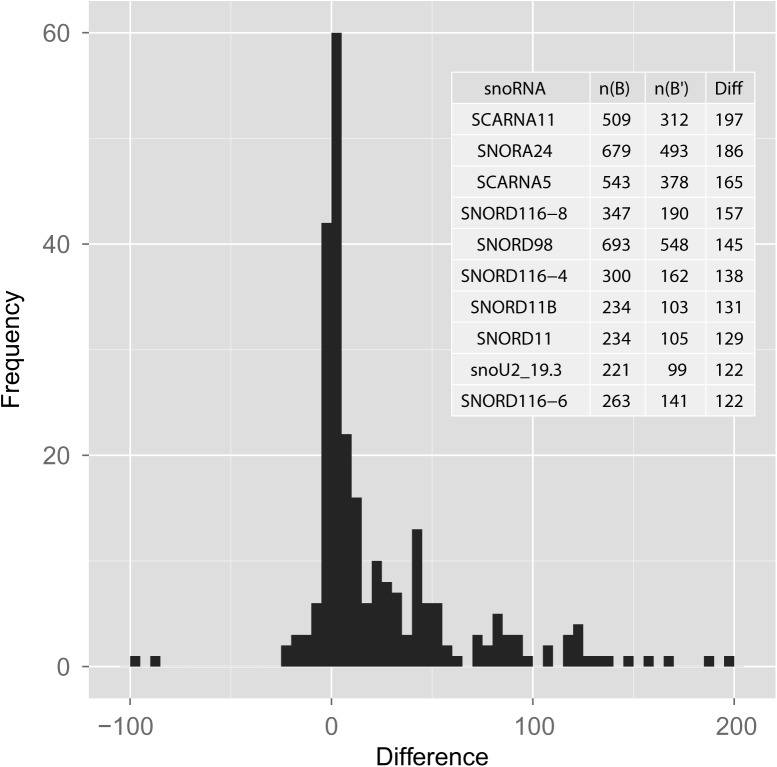
Small nucleolar RNAs have, on average, more conserved complementary targets in intronic segments of protein-coding genes compared with the reverse complements of these segments. The distribution of differences, *D* = *n*(*B*) − *n*(*B*′), where *n*(*B*) and *n*(*B*′) are the number of targets of the same snoRNA on the coding strand and on the opposite strand, respectively. (*Inset*) The top 10 snoRNA with the largest *D*.

The number of conserved complementary pairs in *A* vs. *B* was on average 20% higher than the corresponding figure in *A* vs. *B*′ (*t* = 0.8, *G* = 1, *L* = 10), suggesting that, on average, snoRNAs have an increased capacity to base pair to introns of protein-coding genes. I also noticed that some snoRNAs were more conserved and also had more targets in *B* as well as in *B*′, while others were less conserved and had fewer targets in both sets. To control for the confounding effect of sequence conservation, I considered the difference distribution of the number of targets in the matched sample (Fig. 5). This distribution was skewed to the right and departed significantly from zero (Wilcoxon signed-rank test, *P* = 6 × 10^−^^7^), indicating that most individual snoRNAs have more complementary targets on the coding strand compared with the opposite strand.

**FIGURE 6.**
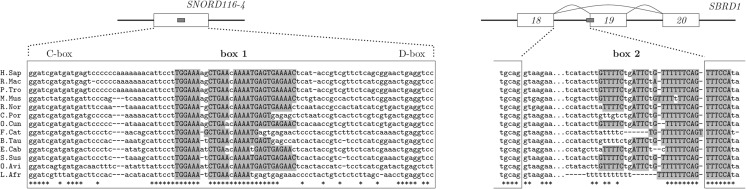
The predicted target of SNORD116-4 snoRNA (chr15:25,304,685–25,304,779) in the human *SBRD1* gene (chr2:45,704,215–45,715,386). Besides C and D boxes, SNORD116-4 contains a conserved sequence box 1 that could potentially mask the acceptor site in SBRD1 and in approximately 280 other mammalian genes.

The distribution of positions of complementary intronic targets was also nonrandom and strongly enriched with targets overlapping acceptor splice sites. An example of such overlapping arrangement is the target of SNORD116-4 in *SRBD1* gene that is shown in Figure 6. Besides C and D boxes, this snoRNA contains a conserved box 1 sequence that is complementary to acceptor site sequences of approximately 280 mammalian genes. Since this enrichment could be due to degeneracy and higher conservation of the intronic sequence upstream of the acceptor site, I compared the average distance of the complementary region to the downstream exon in *A* vs. *B* and in *A* vs. *B*′ and found that the enrichment in targets overlapping acceptor splice sites was, in fact, significant (Wilcoxon signed-rank test, *P* = 2 × 10^−6^). On the other hand, the significance score of each individual RRI was not as convincing as in the case of intramolecular RNA structures (Figs. 3, 4) since both its components were not sufficiently large, *C*_1_ due to highly conserved surrounding sequence and *C*_2_ due to multiple occurrences of the same complement in different genes.

I next asked whether the known complementary targets of HBII-52 were detected (Kishore and Stamm 2006; Kishore et al. 2010). The target in HT_2C_R was correctly identified after replacing the set *B* with the set of exonic segments and reducing the conservation constraints (Supplemental Fig. S5). The targets of HBII-52 in five other genes (Kishore et al. 2010) were not detected due to a combination of factors, including low sequence conservation, a limited number of GT base pairs per *k*-mer, and different seed-matching patterns.

#### LncRNA targets

A similar analysis of 50,000 segments of Gencode v7 lncRNA (set *A*) (Derrien et al. 2012) vs. 200,000 noncoding intronic segments of protein-coding genes (set *B*) also revealed 30% enrichment in the number of targets in the coding strand compared with the opposite strand (*t* = 0.8, *G* = 1, *L* = 13). Sets *A* and *B* were disjoint by construction as lncRNAs that overlap protein-coding genes were initially removed (Supplemental Information). The difference distribution of the matched sample was also skewed and showed a significant departure from zero as in the case of snoRNA targets (Supplemental Fig. S6). The enrichment of lncRNA targets on the coding strand remained positive (21%) after the annotated snoRNAs were removed from the set *A* (the set of lncRNA segments and the set snoRNAs segments were not disjoint), indicating that snoRNA paralogs residing in noncoding RNA genes (Derrien et al. 2012) only partially confound the observed enrichment.

I next asked whether the enrichment of targets on the coding strand would remain when the set of lncRNA segments were replaced by protein-coding segments. To address this, I sampled the set *A*_1_ of segments of protein-coding genes from *B*, as large as the set *A* for lncRNA, and confined the set *B* to *B*_1_ = *B*\*A* in order to keep *A*_1_ and *B*_1_ disjoint (*A* and *B*_1_ are disjoint by construction). Then, I compared the number of targets of *A* in *B*_1_ vs. *A* in *B*_′_^1^, where *B*_′_^1^ as before is the opposite strand control for *B*_1_, with the number of targets of *A*_1_ in *B*_1_ vs. *A*_1_ in *B*_′_^1^. It turned out that lncRNA segments from *A* had on average 25% more targets in the coding strand compared with the opposite strand (average with respect to repeated random sampling of *A*_1_), consistent with the previous figure, while the control protein-coding segments from *A*_1_ had only 7% more targets. The latter figure further decreased to 3% when only segments that were internal to at least one CDS were sampled into *A*_1_, confirming that the enrichment observed for lncRNAs does not arise from artefact in the control procedure.

**FIGURE 7.**
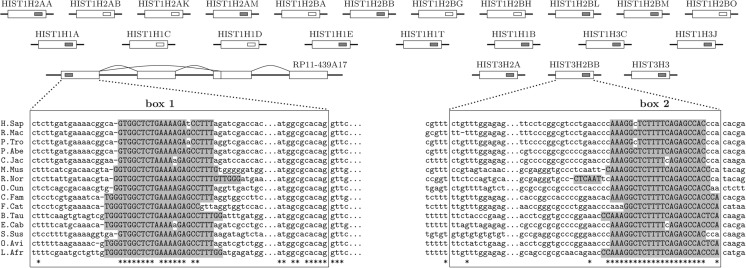
The first exon (chr1:120,876,263–120,905,153) of *RP11-439A17.4* lncRNA (*bottom left*) contains box 1, a conserved sequence that is complementary to box 2 sequence in *HIST3H2BB* gene and also to other similar sequences in 3′ termini of at least 22 mammalian histone genes. Some of the target sequences as well as the reverse complement of box 1 are recognized as MEF-2A binding sites, suggesting coincidental complementarity of transcriptional regulatory elements that are located on the opposite strands of DNA. Annotated (predicted) MEF-2A binding sites are indicated by small gray (white) rectangles.

When doing a similar analysis with noncoding exonic segments, I found an example that could explain the observed enrichment of lncRNA targets in protein-coding genes. This example is the *RP11-439A17.4* lncRNA, which contains box 1 sequence that is complementary to conserved box 2 sequences in 3′ termini of more than 20 mammalian histone genes (Fig. 7). However, a detailed examination revealed that box 2, as well as the reverse complement of box 1, corresponds to MEF-2A (myocyte-specific enhancer factor 2A) TFBS, which occurs on opposite DNA strands. It is also remarkable that *RP11-439A17.4* lncRNA is in the antisense orientation to its neighbor gene *HIST2H2BA*, and therefore, the box 1 sequence within *RP11-439A17.4* is likely to be a transcriptional regulatory element of *HIST2H2BA*. While it is not uninteresting to discover that most mammalian histone genes share the same regulatory motif, I shall conclude that conserved complementarity and RRI in this case are likely to be unrelated. The same argument also applies to all mammalian lncRNAs because many of them are in anti-sense orientation to protein-coding genes.

In summary, although I observe that both snoRNAs and lncRNAs have an increased capacity of base-pairing to introns of protein-coding genes, this observation remains confounded by DNA sequences such as TFBS or other conserved regulatory elements that occur equally likely on either strand of DNA. Evolution maintains these elements conserved and technically complementary for reasons other than base-pairing, which makes them, in principle, indistinguishable from conserved RRIs. Such coincidental complementarity of transcriptional regulatory elements could be the major factor contributing to the false-positive rate in this and in similar previous studies (Raker et al. 2009; Pervouchine et al. 2012).

#### Performance on constrained alignments

Discussed below is the overlap between IRBIS and RNAplex predictions when both programs are confined to the same alignment. Unlike tests in the benchmark section that were done on simulated data, here I sampled 1000 predictions among snoRNA and their targets in intronic noncoding segments of protein-coding genes, applied RNAplex to naïve alignments generated by IRBIS (see Materials and Methods), and considered the base-pairings predicted by RNAplex to be the ground truth. The accuracy, defined as the fraction of base pairs that was predicted by RNAplex among base pairs predicted by IRBIS, was on average 74.6%; namely, most of the base pairs predicted by IRBIS were also predicted by RNAplex. The sensitivity, defined as the fraction of base pairs that was predicted by IRBIS among those predicted by RNAplex, was on average 63.0%. Similar figures of accuracy and sensitivity were observed for mammalian lncRNA targets (65% and 60%, respectively). At that, on these data sets IRBIS ran at least 40 times faster than RNAplex (see next section).

#### Resource requirements

Summarized in Table 2 are memory requirements and run times of IRBIS on a four-core 2.5-GHz CPU with 48 Gb of RAM (serial execution). The assessment was done sequentially on each step: trimming and intramolecular (RSS) and intermolecular RRI prediction (snoRNA and lncRNA targets); the duration of auxiliary steps (data formatting, sequence alignment etc.) was not counted. In all cases, the longest and the most resource-consuming was the step of trimming.

**TABLE 2.**
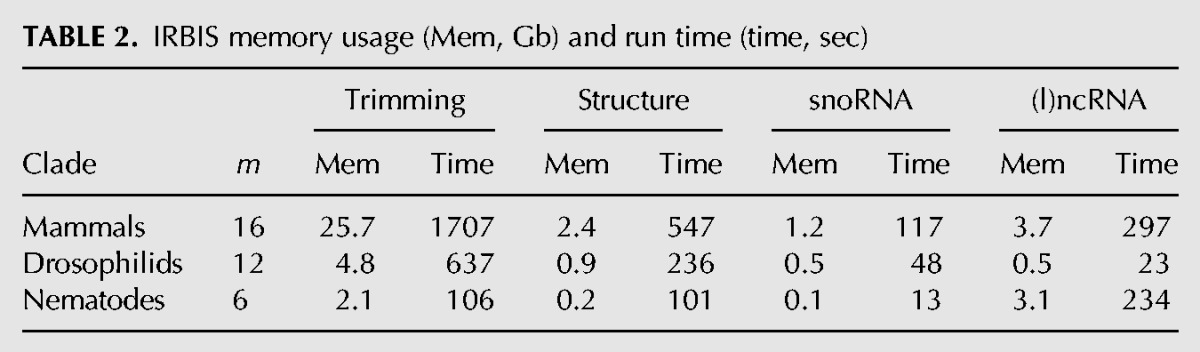
IRBIS memory usage (Mem, Gb) and run time (time, sec)

## DISCUSSION

### Sources of bias

Despite high sensitivity, false-negative predictions may arise from incorrect assignment of orthologous segments, as shown in the example of the *14-3-3*ζ gene. In this case, the chain alignment identified multiple orthologs of the intron between exon 5a and exon 5b, but the syntenic alignment failed to chose the correct projection (Karolchik et al. 2003). Therefore, manual correction of the list of orthologous segments will always be needed in such ambiguous cases. At the same time, the extensive homology between exons and introns in this genomic region indicates that exon 5 cluster of *14-3-3*ζ has evolved through a genomic duplication event that also affected the intron and duplicated one of the arms of the ancestral hairpin, which led to multiple copies of IE1 currently competing for the same IEa sequence. This leads to an interesting hypothesis that genomic duplications copying one of the complementary boxes could be a generic evolutionary mechanism for generating structural RNA switches that control mutually exclusive exon skipping.

The FDR estimation procedure measures how many conserved complementary regions are located in introns of protein-coding genes compared with conserved complements between introns of different genes. Whether or not the latter are biologically functional, I find a significant enrichment for intramolecular RNA structures and FDR figures that is consistent with the previous report (Pervouchine et al. 2012). Additionally, the current analysis of the intermolecular RRI and, in particular, the example of *RP11-439A17.4* lncRNA targets (Fig. 7) suggest that a substantial fraction of false-positive predictions comes from sequence motifs that occur on either strand of DNA and are maintained by evolution for purposes other than base-pairing. These conserved DNA elements are, in principle, indistinguishable from the conserved base-pairings in RNA, therefore confounding all such analyses.

Considerable discrepancies in sequence alignments that are currently produced by IRBIS arise from the fact that only one pair of conserved complementary regions is reported per pair of segments. Some segments contain a single such match (Figs. 3 and 4), while others may contain multiple distinct matches, and consequently, if the naïve procedure picks up seeds from different complementary pairs, it will produce a wrong alignment. This seeming limitation will be addressed in future implementations. It contains a nontrivial task of predicting joint RNA structure and alignment given the “skeleton” of conserved complementary regions, which is beyond the focus of this report.

### First fold or first align?

The first-fold-then-align paradigm in comparative RNA structure prediction is frequently implemented in combination with the thermodynamic model (Mathews et al. 1999). While this model has been undoubtedly tested as most accurate for short molecules, its scalability to large eukaryotic RNAs is limited, for instance, by the confounding effects of RNA–protein interactions which increasingly contribute to the free energy with growing length. A bigger limitation, however, has to do with the optimization of the free energy by dynamic programming, which has to ignore pseudoknots in order to be computationally possible. As a result, only a small corner of the structure space is explored (essentially, long-range RNA structures are simply ignored). This leads to a strong bias toward local base-pairings that also increases with sequence length. A subset of parameters still can be used as a scoring schema, but in the case of long complementary regions, its advantage over other scoring schemas is not evident. The approach taken by IRBIS is to control for the GC content, which is largely equivalent to thresholding by the free energy.

Many conserved complementary boxes can also be found directly in blocks of MSA at UCSC Genome Browser (Karolchik et al. 2003). While this is certainly true for some genes, the alignment-free approach is essential even in well-conserved genomic regions. For example, the MSA in the *msn* gene (Supplemental File 1, Fig. 2) at UCSC lacks some of the sequences due to misalignment. Nevertheless, a novel direction in which MSA-based methods could next develop is the exhaustive genome-wide analysis of short, well-conserved intronic motifs. In the majority of studied cases, the evolutionary constraints were so high that the complementary motifs remained almost unchanged during large evolutionary distances such as, for instance, mammalian radiation (Raker et al. 2009; Pervouchine et al. 2012; Li and Breaker 2013). Therefore, the computational tools that make use of compensatory base changes, in fact, cannot use this information because mutations in base-paired regions do not occur frequently enough. In contrast, mutations do occur in the adjacent regions that are not under selection, and therefore, one should expect gaining more statistical power by observing conserved complementary motifs in nonconserved background than by tracking rarely occurring compensatory base changes.

### Concluding remarks

Most eukaryotic protein-coding genes are organized by the nature in a discontinuous way so that well-conserved exons alternate with less-conserved introns. Exons evolve under protein-coding constraints, have higher sequence identity, and often can be aligned at the nucleotide level. In contrast, introns are less constrained, have low sequence identity, and usually do not align well. The trick of this approach is in extending the relationship of orthology from exons to interjacent introns by synteny without actually creating the alignment. In this light, the collection of unaligned genomic segments in Figure 1 represents a very general setting.

Although conserved RNA structures in exonic segments are efficiently analyzed by MSA-based methods, protein-coding constraints often mask conservation patterns that arise in exons on top of the genetic code. The analysis of intronic RNA structures, in contrast, gives statistically very significant patterns: conserved complementary “islands” contrasting with nonconserved intronic background. We therefore arrive at the dichotomy in which the conserved genomic blocks are analyzed by MSA-based methods, while the divergent blocks are analyzed by alignment-free methods such as one described here. At that, the structure-based sequence homology induces an alignment in intronic sequences, which narrows the search to shorter segments where the entire procedure can be applied recursively. This strategy in combination with other recent approaches (Will et al. 2013) has many important implications to whole-genome realignments.

## MATERIALS AND METHODS

### Data preparation

IRBIS contains a number of preprocessing steps, starting from genomic sequences and pairwise sequence alignments (Supplemental Fig. S1). The default segmentation is the one induced by exon boundaries. Segments are classified into exonic vs. intronic and coding vs. noncoding as explained in the Supplemental Methods. One species in the clade (e.g., human in placental mammals) is chosen as a reference, and the orthologous segments are lifted over uniquely from the reference genome to the target genomes using pairwise BLASTz whole-genome alignments (Supplemental Fig. S2; Karolchik et al. 2003). There is an upper limit on the segment length such that if a segment contains more than *M* nucleotides, then only the first *M*/2 and the last *M*/2 nucleotides are considered and the middle part of the segment is discarded (the purpose of this limit is to prevent memory overflow by very long segments that may be occasionally generated by liftOver). GENCODE.v7, BDGP5.25.64, and WS220.65 annotations and GRCh37, BDGP5, and WS220 genome assemblies were used for *Homo sapiens*, *Drosophila melanogaster*, and *Caenorhabditis elegans*, respectively. Genomes references and their corrected Gerstein-Sonnhammer-Chothia weights (Gerstein et al. 1994) are listed in Supplemental Table S1. The detailed protocol on data processing is given in Supplemental Methods.

### Hashing

For ease in description, I first focus on *k*-mers without gaps. In each species *i*, I create a hash table which appoints to each *k*-mer ω the array *H*_*i*_(ω) of ordered pairs such that (*j*, *p*) ∈ *H*_*i*_(ω) if and only if ω occurs at the position *p* of *s*_*ij*_. We may assume that *H*_*i*_ is being populated sequentially by reading *s*_*ij*_ from left to right and that all segments were initially sorted by increasing *j*. Then, the array *H*_*i*_(ω) will be automatically sorted in lexicographic order, namely, (*j*, *p*) ≤ (*j*′, *p*′) if *j* < *j*′ or *j* = *j*′ and *p* ≤ *p*′. In what follows, *H*_*i*_(ω) shall be regarded as a partially ordered finite set with respect to an abstract partial order ≤. From the perspectives of time and storage, a feasible range of value of *k* is from 8–11 nt (the default value is 8). However, the value of *k* does not serve as a thermodynamic cutoff since in what follows I aggregate overlapping *k*-mers and use a different threshold *L* (minimum length of complementary region, see below).

We are interested in finding *k*-mers that occur in multiple *s*_*ij*_ for the same *j* (in what follows I will refer to such *k*-mers as *conserved*). To do this, I introduce a “position-forgetting” relation by setting (*j*_1_, *p*_1_) ≃ (*j*_2_, *p*_2_) whenever *j*_1_ = *j*_2_. It defines an equivalence relation between elements of *H*_*i*_(ω) in different *i* so that the most conserved *k*-mers correspond to the largest equivalence classes. Since the conserved *k*-mers are detected regardless of their position in *s*_*ij*_, a stronger form of ≃, where (*j*_1_, *p*_1_) ≃ (*j*_2_, *p*_2_) whenever *j*_1_ = *j*_2_ and |*p*_2_ − *p*_1_| < Δ, can be used instead to take into account distance divergence between *k*-mers, although unaligned sequences cannot be adequately compared by position.

The equivalence relation ≃ is too strict in a sense that it assumes exact sequence conservation. As a generalization, I consider gapped-seed hash tables, in which *k*-mers are allowed to have gaps (Supplemental Fig. S3). Gaps serve two purposes: (1) they model short internal loops in RNA structures, and (2) they allow for a small number of mutations in conserved regions. Although asymmetric gapped seeds were shown to perform better in the framework of lossy filtration for sequence alignment (Ma et al. 2002), here I cluster and extend overlapping k-mers so that the advantages of asymmetric seeds cannot be fully used. Hence, I assume that the gap is always in the middle of the *k*-mer. The gapped method is explained in more detail in the Supplemental Methods.

### Trimming

**FIGURE 8.**
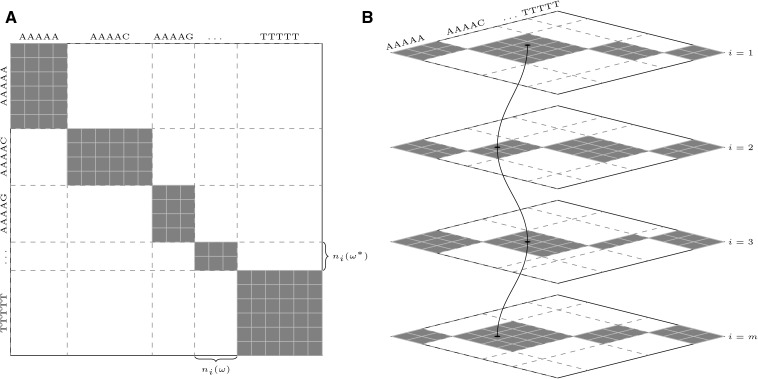
(*A*) A diagram exemplifying the helix space. Horizontal and vertical axes correspond to the hash tables *H*_*i*_ and *H*_i_^*^, respectively; widths and heights of the gray rectangles are *n*_*i*_(ω) and *n*_*i*_(ω*), respectively. The total gray area represents the number of (ordered) pairwise combinations of complementary *k*-mers. (*B*) Several diagrams as in *A* need to be parsed simultaneously in order to find conserved complementary *k*-mers. Pairs equivalent under ≃ are connected by a path.

In order to count all perfect Watson-Crick complementary helices of length *k* in species *i*, consider the diagram in Figure 8A. Along the *x*-axis I list all *k*-mers, and for each *k*-mer ω, I list all its occurrences in *s*_*ij*_, namely, the elements of *H*_*i*_(ω). Similarly, along the *y*-axis I list all *k*-mers, and for each ω, I list all occurrences of ω*, namely, the elements of *H*_i_^*^(ω) = *H*_*i*_(ω^*^), where ω* is the reverse complement of ω. Then, by construction, each little square in the gray area corresponds to a perfect complementary helix of length *k*. If ω occurs *n*_*i*_(ω) times and ω* occurs *n*_*i*_(ω*) times, then there are *n*_*i*_(ω) · *n*_i_^*^(ω) such helices.

The largest gray area occurs when one *n*_*i*_(ω) is large for some ω, for example, when *s*_*ij*_ consist of poly-nucleotides (e.g., poly-A and poly-T). The smallest gray area occurs when gray rectangles are squares of approximately the same size, but even in this best case, the required storage is impractically large (Supplemental Material). In order to find complementary *k*-mers that are conserved, we need to analyze such diagrams for different *i* simultaneously (Fig. 8B). Therefore, a prefiltering step is needed to reduce the size of the gray area. Such prefiltering, called *trimming*, is described in detail in the Supplemental Material. Trimming makes use of the fact that *H*_*i*_(ω) is an ordered array, and therefore, the elements of *H*_*i*_(ω) can be quickly compared to discard nonconserved *k*-mers (Supplemental Fig. S4). Trimming takes as an input a collection of hash tables *H*_*i*_(ω) and returns a collection of sparcified hash tables, one in which (*j*, *p*) ∈ *H*_*i*_(ω) is retained if and only if the sum of weights of the corresponding species is greater than threshold *t*_0_ (Table 1).

### Cartesian product and intersection

After trimming, *H*_*i*_ and *H*_i_^*^ contain locations of conserved *k*-mers only. However, it will not be enough just to report all pairwise combinations of ω and ω* because, for instance, if ω is found in species 1, 3, and 5, while ω* is found in species 2, 4, and 6, then the two together may not be present in the same species (Fig. 1). To account for this, I apply the trimming procedure to the hash table *P*_*i*_ defined by *P*_*i*_(ω) = *H*_*i*_(ω) × *H*_i_^*^(ω), where × is Cartesian product. *P*_*i*_(ω) carries a canonical lexicographic order, one in which (α_1_, β_1_) ⪯ (α_2_, β_2_) whenever α_1_ < α_2_ or α_1_ = α_2_ and β_1_ ≤ β_2_. Hence, the same trimming routine (Supplemental Material) can be applied to *P*_*i*_ with respect to the new order ⪯, possibly at a different threshold *t*.

The rationale behind using a different threshold *t* for the intersection is that the most time-consuming step is the creation and processing of large hash tables. Therefore, it is convenient to use an intermediate threshold *t*_0_ to initially trim hash tables and store them as meta-data. The meta-data can then be loaded and quickly trimmed again, if necessary, at a higher threshold *t* > *t*_0_ according to problem-specific needs (Supplemental Fig. S1).

The array *P*_*i*_(ω) lists all pairwise combinations of the occurrences of ω with the occurrences of its exact reverse complement. I introduce wobble base pairs by modifying the definition of the reverse complement so that ω* is not a single *k*-mer but a collection of *k*-mers that is complementary to ω with a small number of GU base pairs. This change affects only *H*_i_^*^(ω), which is now redefined as

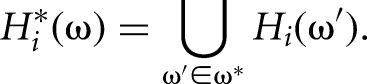

In some applications, it is needed to limit all-to-all combinations to some predefined subset of combinations (e.g., only miRNAs vs. their targets). This is achieved by (1) selecting two (not necessarily disjoint) subsets of segments, *A* and *B*, called the left and right set, respectively; building two separate hash tables, *H*_*A*,*i*_ and *H*_*B*,*i*_, one for each set; and setting *P*_*i*_(ω) = *H*_*A*,*i*_(ω) × *H*_*B*,i_^*^(ω); and (2) specifying a binary relation ℛ ⊆ *A* × *B* and taking *P*_*i*_(ω) as a subset relative to ℛ; namely, (α, β) ∈ *P*_*i*_(ω) only if α = (*j*, *p*), β = (*j*′, *p*′), and (*j*, *j*′) ∈ ℛ. Since *A* and *B* may intersect, the same pair of complementary *k*-mers may be reported twice. To prevent this, there is an option to report only (α, β) ∈ *P*_*i*_(ω) such that α < β. All these options are listed in Table 1.

### Post-processing

The output of the above steps is the set of arrays, *P*_*i*_(ω), whose elements are pairs (α, β), where α = (*j*, *p*), β = (*j*′, *p*′), *p* is the position of ω in *s*_*ij*_, and *p*′ is the position of ω* in *s*_*ij*__′_. It often happens that some *k*-mers overlap each other, forming complementary stretches of more than *k* nucleotides. Therefore, a post-processing step is needed to identify and merge such overlapping *k*-mers. It is achieved by sorting quadruples (*j*, *p*, *j*′, *p*′) lexicographically by *j*, *j*′, *p*, and *p*′ (in this order); then, the overlapping *k*-mers must occur sequentially in the sorted list, and the longest clusters can be identified, for instance, by dynamic programming.

In principle, these clusters contain exhaustive information about conserved complementary regions. However, a human-readable output assumes choosing a unique combination of such clusters based on some criteria of optimality and building the respective structure-based sequence alignment. This problem concerns a very different aspect of simultaneous folding and alignment and, in general, could be as complex as Sankoff algorithm (Sankoff 1985). Here I implement the minimal formulation (referred to as *naïve*) by selecting the longest complementary region (or the first occurring such region, if there are many) when its length is greater or equal to some threshold value *L*, separately aligning complementary regions and intervals between them, and merging the resulting alignments. Consequently, the naïve alignment reports only one pair of conserved complementary regions per segment pair, while more accurate analyses would predict multiple such regions (see Discussion).

The alignment of complementary regions and intervals between them was done by MUSCLE (Edgar 2004a,b). Sequences >150 nt were trimmed (by removing sufficiently many nucleotides in the middle) prior to the alignment. The merged alignments were represented graphically by custom libraries based on L^A^T_E_X and TikZ.

## SUPPLEMENTAL MATERIAL

Supplemental material is available for this article.

## Supplementary Material

Supplemental Material
