# Supplementary material for: IRBIS: a systematic search for conserved complementarity
Source: RNA. 2014 Oct;20(10):1519–31. doi: 10.1261/rna.045088.114 (PMC4174434; doi:10.1261/rna.045088.114)
Supplement: Supplemental Material [file supp_20_10_1519__index.html]

IRBIS: a systematic search for conserved complementarity — IRBIS: a systematic search for conserved complementarity — Supplemental Material 

# IRBIS: a systematic search for conserved complementarity

## Supplemental Material

**Files in this Data Supplement:**

- Supp Material.pdf
