# Supplementary material for: IRBIS: a systematic search for conserved complementarity
Source: RNA. 2014 Oct;20(10):1519–31. doi: 10.1261/rna.045088.114 (PMC4174434; doi:10.1261/rna.045088.114)
Supplement: Supplemental Material [file supp_045088.114_SuppMaterial.pdf]

IRBIS: a systematic search for conserved complementarity

Supplementary Material

Contents

1 Methods **2**

1.1 Data preparation 2

1.2 Rewiring control 4

1.3 Hash tables with gapped seeds 4

1.4 The helix space 5

1.5 Trimming 5

1.6 Estimation of statistical significance 6

2 Supplementary Figures and Tables **7**

3 Supplementary Files **15**

1 Methods

1.1 Data preparation

In this section we outline the methodology used to build the input data set for IRBIS pipeline.

Genomic sequences of placental mammals, drosophilids, and nematodes (Table S1) were downloaded from UCSC Genome Browser website (Karolchik *et al.* , 2003). Gerstein-Sonnhammer-Chothia weights were computed based on mya-divergence between species (Gerstein *et al.* , 1994). At that, weights outside of the interquartile range were replaced by the respective quartiles (the identifiers and weights in Table S1). The subsequent analyses were performed separately for mammals, drosophilids, and nematodes as explained below (see also flowchart in Figure S1). One species in the clade (e.g., human in placental mammals) was chosen as a reference. Segments were lifted over from the reference genome to the other genomes and unique projection were selected (explained below).

The following genomic annotations were used: GENCODE.v7 for *H. sapiens*, BDGP5.25.64 for *D. melanogaster*, and WS220.65 for *C. elegans*. These three annotations were used as reference transcript models, i.e., the existing annotations in the other species were not taken into account. In what follows, *H. sapiens*, *D. melanogaster*, and *C. elegans* will be called reference species for each of the three groups of organisms.

The collection of exons in the reference annotation was used to build a list of exon boundaries, each identified by chromosome, position, and strand (CPS). Next, orthologs of exon boundaries in each of the other species were identified by a custom liftOver procedure (`map_single` utility) using pairwise whole-genome chain alignments (Raker *et al.* , 2009; Pervouchine *et al.* , 2012). In this procedure an exon boundary of the reference species was projected to a target species (generally, more than once) each time it was found in an aligned block of a chain. The unique projections were selected by a procedure implementing

maximum synteny on per-gene basis as explained in Figure S2 (`unique_match` utility). That is, each segment (i.e., an interval between two consecutive exon boundaries) in the reference species was projected to other species by assigning an ortholog to a segment if and only if the corresponding boundaries were uniquely matched.

Segments inherit annotated gene identifiers so that one segment may belong to multiple overlapping genes. We define a segment in the reference genome to be *covered* by a transcript if the segment is located between the transcript's start and end. Then each segment falls into one of the four categories: (1) *exonic*, (2) *alternative exonic*, (3) *intronic*, (4) *other*, if (1) it belongs as an exon to all covering transcripts, (2) it belongs as an exon to at least one covering transcript and doesn't fall into the previous category, (3) it belongs as an intron to all covering transcripts, and (4) none of the above options, respectively (the fourth category has to do with the genes that have disconnected transcript graphs).

Independently of this classification, each segment also falls into one of the two coding categories, *(1)* coding, *(2)* non-coding, if (1) it intersects with at least one annotated CDS, and (2) otherwise. Additionally, each segment inherits gene type attribute of the gene to which it belongs. This splits all segments by biotype into protein-coding, long non-coding lncRNAs (for GENCODE.v7 it is lincRNA + processed transcript biotypes except for segments overlapping protein-coding genes on either strand), snoRNAs (except for segments overlapping protein-coding genes), and all other segments.

This procedure gave rise to a collection of orthologous segments, of which we study in most detail non-coding intronic segments of protein-coding genes, long non-coding RNAs (lncRNA), and snoRNAs. Genomic sequences of orthologous segments were retrieved and converted to metafiles by the `trim` utility (see flowchart in Figure S1). The metafiles were then passed to `irbis` utility in different combinations along with set A, set B, and the relation $\mathcal{R}$. The resulting lists of clusters of conserved complementary k-mers were passed to the alignment routines `tab2maf` and `tab2pdf` to create human-readable output in pdf format.

The described pipeline (Figure S1) was executed using GNU `make` utility. More information on each individual step can be found in the manual that comes with the pakage.

1.2 Rewiring control

The re-wiring control procedure creates hybrid transcripts that consist of segments that belong to different genes. It is essentially equivalent to shuffling gene identifiers of the segments. To do this, we stratified segments into 500 blocks (10 levels of the GC content and 50 levels of the conservation score) and randomly shuffled segment identifiers within each block. Next, the relation $\mathcal{R}$ such that $(j, j') \in \mathcal{R}$ if and only if j and j' belong to the same gene was transformed into relation $\mathcal{R}'$, in which the first component was as in $\mathcal{R}$ and the second component was defined by the shuffling.

1.3 Hash tables with gapped seeds

The implementation of gapped-seed hash tables, in which k-mers are allowed to have gaps, in which k-mers are allowed to have gaps, serves two purposes:

1. Gaps model short internal loops in RNA structures;
2. Gaps allow for a small number of mismatches in conserved complementary regions.

The gapped-seed method is different from the ungapped method in the stage of construction of the hash table and and also in the post-processing stage.

Gapped seeds are defined by the pattern m-g-m (match-gap-match) as follows. Consider all pairs of m-mers that are separated by $l \leq g$ nucleotides, concatenate them by removing the inner l and keeping the resting $k = 2m$ nucleotides, and use the resulting k-mer to construct the hash table as before. However, in order to locate the gapped seed in the original sequence, $H_i(\omega)$ must now contain ordered triples (j, p, l), where j and p are as before and l is the size of the gap. Assuming that s_{ij} is being scanned successfully from left

to right, the triples (j, p, l) automatically occur the hash table $H_i(\omega)$ sorted in lexicographic order. This process is exemplified by the schema in Figure S3.

1.4 The helix space

In this section we will estimate C_i, the size of the helix space, which is defined to be the total number of perfect Watson-Crick complementary helices of length k in all segments of species i (gaps not allowed). Denote by $n_i(\omega)$ the number of elements in $H_i(\omega)$ and define the hash table H_i^* by the rule $H_i^*(\omega) = H_i(\omega^*)$, where ω^* is the reverse complement of ω. Since ω occurs in $n_i(\omega)$ positions and ω^* occurs in $n_i(\omega^*)$ positions, they can form $n_i(\omega) \cdot n_i(\omega^*)$ perfect complementary helices of length k as illustrated by gray rectangles in Figure 8. Thus,

$$C_i = \sum_{\omega} n_i(\omega) \cdot n_i(\omega^*).$$

The largest gray area occurs when s_{ij} consist of poly-nucleotides (e.g., poly-A and poly-T) for all j and in that case $C_i = N^2/2$, where

$$N = \sum_{\omega} n_i(\omega) = \sum_{\omega} n_i(\omega^*).$$

The smallest gray area occurs when all the rectangles are squares of the same size, i.e., when $n_i(\omega) = n_i(\omega^*) = N/4^k$ for all ω; then, $C_i = N^2/4^k$. A brief estimation of these upper and lower bounds shows that the average storage for C_i in the case of mammalian genomes is on the order of hundreds of gigabytes (120 Gb for human genome), but it could in fact be much larger depending on the repetitive content.

In practice, H_i, H_i^*, and all pairwise combinations of their elements have to be checked, which can be problematic for big data sets. The size of hash tables can be reduced by selecting a reasonable segment size M and discarding repetitive or AT-rich k-mers. The following procedure called trimming is applied to further reduce the size of H_i by removing *a priori* non-conserved k-mers from consideration.

1.5 Trimming

Trimming is carried out sequentially for each k-mer ω as follows. Denote $n_i(\omega)$ for simplicity by n_i, consider the array $H_i(\omega) = (\alpha_{i\,1}, \alpha_{i\,2}, \ldots, \alpha_{i\,n_i})$, initialize $b_i = 1$ for all $i = 1 \ldots m$, and compute $\alpha = (j,p) = \min\{\alpha_{i\,b_i}\}$ with respect to $\leq$ over i. The set $S = \{i|\alpha_{i\,b_i} \simeq \alpha\}$ is the set of species which contain ω in s_{ij} for the given j. Since $H_i(\omega)$ are sorted, α is also the global minimum of all $H_i(\omega)$ and, therefore, all occurrences of ω in orthologous segments are already counted for the given j. The element $\alpha_{i\,b_i}$ is kept in $H_i(\omega)$ only if $\theta \geq t_1$ for some threshold t_1, where

$$\theta = \sum_{i \in S} f_i, \qquad (1)$$

where f_i are species weights; otherwise, $\alpha_{i\,b_i}$ is discarded for all $i \in S$. The loop is closed by incrementing b_i for all $i \in S$, and repeating all steps from the computation of α until $b_i = n_i$ for all i. This procedure is illustrated in Figure S4.

Since b_i run over the elements of H_i in increasing order, the time complexity is linear with respect to the combined number of entries in all H_i and, therefore, is also linear with respect to the combined length of all sequences.

1.6 Estimation of statistical significance

The p-value for a pair of conserved complementary regions is computed with respect to two distinct null hypotheses. The conservation test evaluates how unlikely it is to find a stretch of conserved nucleotides, as large as the one observed, in a segment with the given the average nucleotide conservation rate. If a pair of segments (j, j') contains conserved complementary regions, both of length K, then the negative logarithm of p-value is given by

$$C_1 = -\theta \left(K \log \gamma_j + K \log \gamma_{j'} + \lambda_j + \lambda_{j'} \right), \qquad (2)$$

where γ_j and $\gamma_{j'}$ are the respective nucleotide conservation rates, λ_j and $\lambda_{j'}$ are factors related to segment lengths, and θ is defined in the main text. Although a MSA is not available, it is still possible to estimate γ_j and $\gamma_{j'}$ since k-mers themselves provide a measure of local sequence homology (Edgar, 2004a). They

can be found from the fractional common k-mer count or by comparing $H_i(\omega)$ before and after trimming. Note that dinucleotide-preserving sequence shuffling is not applicable without MSA.

The complementarity test estimates how "rare" is the observed pair of complementary regions. Namely, for each ω we define $p_i(\omega)$ to be the percentile of $n_i(\omega) \cdot n_i(\omega^*)$ in the distribution of such non-zero products over all k-mers in H_i and let p_i be the smallest $p_i(\omega)$ among the k-mers in the observed complementary regions. Then the negative logarithm of p-value is given by

$$C_2 = -\sum_{i \in S} f_i \log p_i. \quad (3)$$

The value $10^{-N(C_1+C_2)}$, where N is the number of trials to account for multiple hypothesis testing, is used as a combined p-value to rank the individual predictions. Additionally, a linear combination of C_1 and C_2 can be used to limit the output according to the rule $N(C_1 + C_2) \geq C$, where C is the significance threshold.

2 Supplementary Figures and Tables

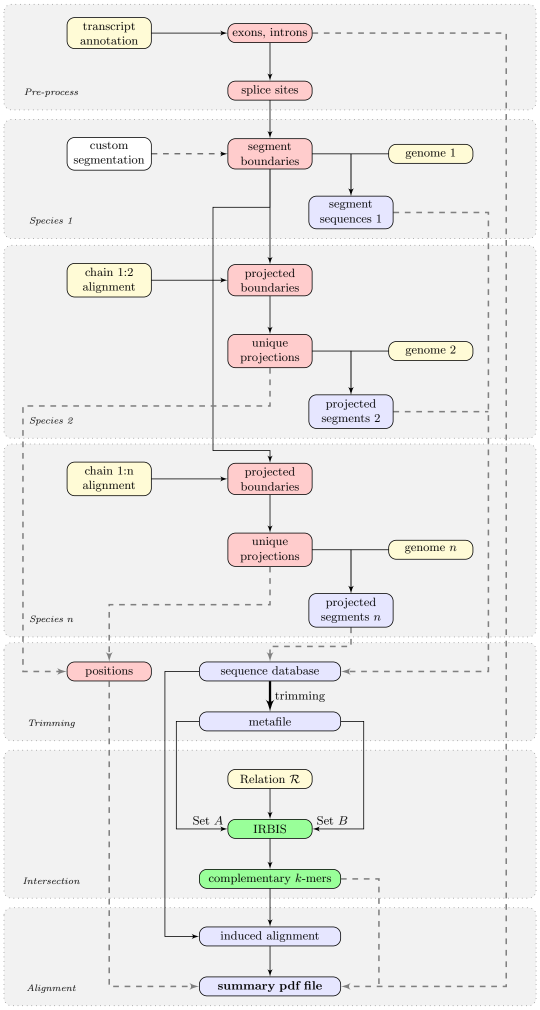

Figure S1: Data processing flowchart.

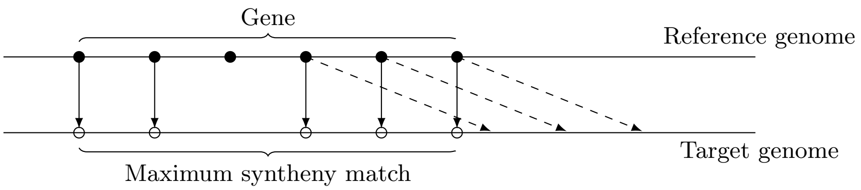

Figure S2: Lift-over. Non-ambiguous orthologous exon boundaries were selected by a dynamic programming routine that maximizes the length of the aligned region containing exon boundaries given that the length of each interval between two consecutive exon boundaries is changed by not more than 50% or by less than 5000 nucleotides (up to four consecutive exon boundaries can be ignored in this procedure).

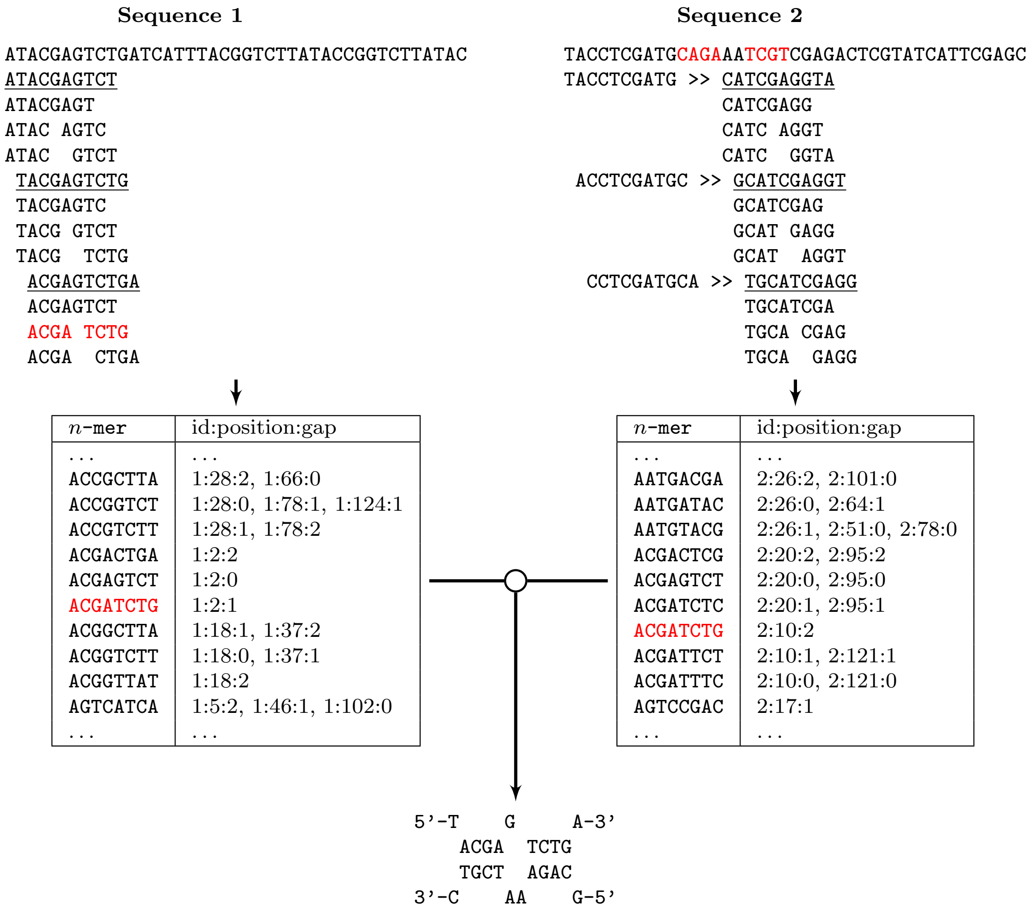

Figure S3: The construction of a gapped-seed hash table for $k = 8$ and $g = 2$. The sequence is cut into $(k+g)$-mers and each $(k+g)$-mer gives rise to $g+1$ k-mers by keeping the first $k/2$ characters fixed and taking the resting $k/2$ characters with a gap of l nucleotides, $0 \le l \le g$. The elements of the hash table are triples (j, p, l), where j is the sequence identifier, p is the position of the first nucleotide of the k-mer, and l is the size of the gap. The RNA structure shown on the bottom can be detected when two such hash tables are intersected.

k-mer	species	segment:position
...		...
AAAAG	1	1:100, **2:100**, 7:200, ...
	2	**2:150**, 4:200, 6:100, ...
		...
	m	**2:120**, 7:300, 8:400, ...
...		...

Figure S4: An example of hash table trimming for the k-mer AAAAG. The first minimum element is 1:100, $S = \{1\}$; sum of weights doesn't reach the threshold; it is discarded. The second minimum element is 2:100, $S = \{1, 2 \ldots, m\}$, sum of weights is at maximum and all entries shown in boldface are retained.

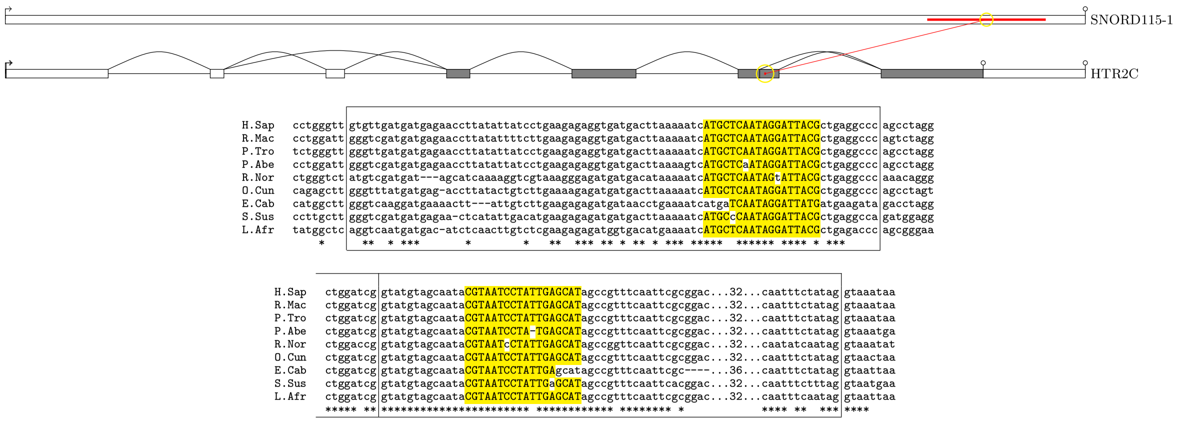

Figure S5: The predicted complementarity region between snoRNA HBII-52 and exon V of 5-HT$_{2C}$R.

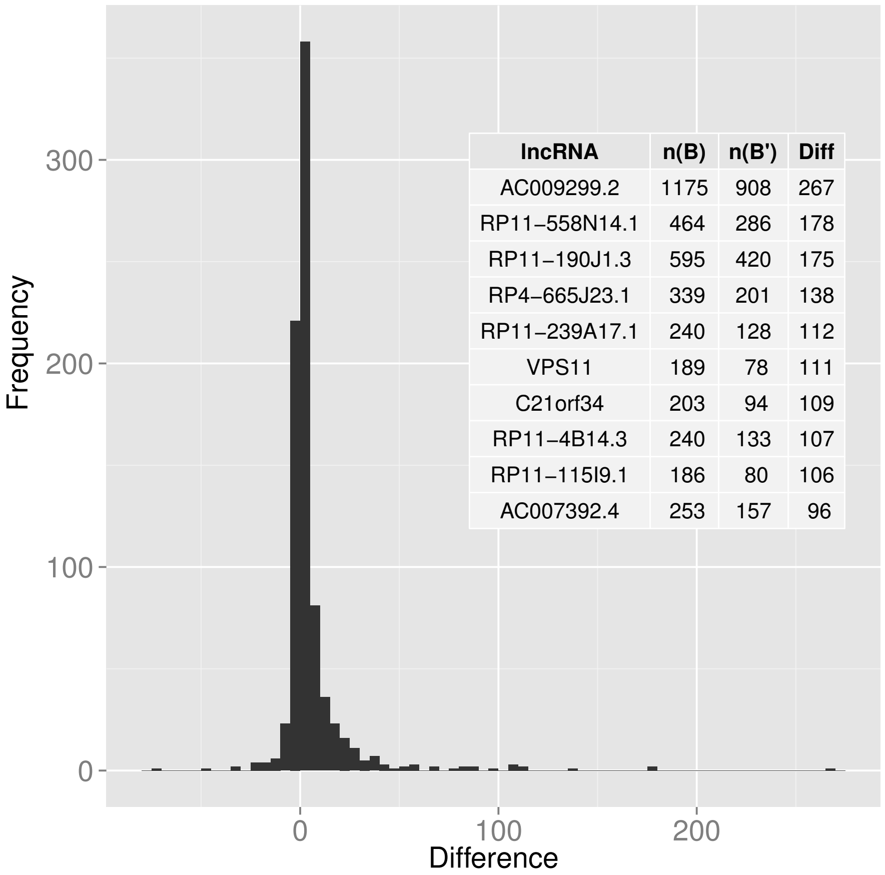

lncRNA	n(B)	n(B')	Diff
AC009299.2	1175	908	267
RP11−558N14.1	464	286	178
RP11−190J1.3	595	420	175
RP4−665J23.1	339	201	138
RP11−239A17.1	240	128	112
VPS11	189	78	111
C21orf34	203	94	109
RP11−4B14.3	240	133	107
RP11−115I9.1	186	80	106
AC007392.4	253	157	96

Figure S6: The distribution of differences, $D = n(B) - n(B')$, where $n(B)$ and $n(B')$ are the number of targets on the coding strand and on the opposite strand for the same lncRNA. Inset: the top 10 lncRNAs with the largest D.

Table S1: Genomes, species and their weights.

Species	Assembly	Weight	Species	Assembly	Weight
Drosophila ananassae	droAna3	8.11	Felis catus	felCat4	50.60
Drosophila erecta	droEre2	6.76	Homo sapiens	homSap19	35.50
Drosophila grimshawi	droGri2	9.64	Loxodonta africana	loxAfr3	75.40
Drosophila melanogaster	droMel3	8.24	Mus musculus	musMus9	43.50
Drosophila mojavensis	droMoj3	8.03	Oryctolagus cuniculus	oryCun2	83.90
Drosophila persimilis	droPer1	8.09	Ovis aries	oviAri1	34.51
Drosophila pseudoobscura	droPse4	7.12	Pan trogloditis	panTro3	27.20
Drosophila sechellia	droSec1	6.38	Pongo pugmeus abelii	ponAbe2	37.10
Drosophila simulans	droSim1	9.29	Rattus norvegicus	ratNor4	43.50
Drosophila virilis	droVir3	7.58	Macaca mulatta	rheMac2	27.20
Drosophila willstoni	droWil1	8.59	Sus scrofa	susScr2	43.14
Drosophila yakuba	droYak2	7.59	Caenorhabditis brigsae	caeBri3	1.0
Bos taurus	bosTau4	45.80	Caenorhabditis brenneri	caeBre2	1.0
Callithrix jacchus	calJac3	32.90	Caenorhabditis elegans	caeEle6	1.0
Canis familiaris	canFam2	50.60	Caenorhabditis japonica	caeJap1	1.0
Cavia porcellus	cavPor3	80.90	Caenorhabditis remmanei	caeRem3	1.0
Equus caballus	equCab2	45.80	Pristionchus pacificus	priPac1	1.0

3 Supplementary Files

Supplementary files are found at http://genome.crg.es/∼dmitri/irbis/supplementary/. Each file contains a multiple sequence alignment induced by conserved complementary regions (see main text). Alignments are organized on one-per-page basis so that, for instance, Figure 111 is found on page 111.

**Intra-molecular (secondary) RNA structure**	
Drosophilids	SuppFile1
Placental mammals	SuppFile2
Nematodes	SuppFile3

**SnoRNA targets**	
Drosophilids	SuppFile4
Placental mammals	SuppFile5
Nematodes	SuppFile6

**Specific examples**	
Targets of RP11-439A17.4 lncRNA	SuppFile7
